# Compassion Scale: factor structure and scale validation in Hong Kong adolescents

**DOI:** 10.3389/fpsyg.2025.1508402

**Published:** 2025-02-26

**Authors:** Steven Sek-yum Ngai, Chau-kiu Cheung, Yuen-hang Ng, Hao-yi Guo, Han-lei Du, Chen Chen, Laing-ming Wong, Qiu-shi Zhou, Wing-tsam Pang

**Affiliations:** ^1^Department of Social Work, The Chinese University of Hong Kong, Hong Kong, Hong Kong SAR, China; ^2^Department of Social and Behavioral Sciences, City University of Hong Kong, Hong Kong, Hong Kong SAR, China

**Keywords:** compassion toward others, adolescents, scale validation, factor structure, Hong Kong

## Abstract

**Introduction:**

This study aimed to validate the 16-item Compassion Scale (CS) for use with Hong Kong adolescents. 1,193 secondary school students in grades 7 to 11 (*M* = 13.80 years, 43.3% female) completed the questionnaire survey.

**Methods:**

We used EFA and CFA to examine the factor structure of the CS and assessed its convergent and discriminant validity through CR, AVE, AIC, and BIC calculations. We also evaluated the concurrent validity by analysing partial correlations between the CS and its subscales with social connectedness and self-efficacy. In addition, we employed multigroup analysis to determine the model fit of the CS across demographic subgroups.

**Results:**

Factor analyses showed a three-factor structure combining mindfulness and kindness into one factor that we named benevolence, together with common humanity and indifference. Satisfactory model fit was found in different subgroups across age, gender, grade, and school type. Findings suggested that girls, on average, showed a higher level of compassionate concern for others than boys, and younger adolescents were more compassionate than their older counterparts. The CS and its subscales were significantly and positively correlated with social connectedness and self-efficacy, supporting concurrent validity.

**Discussion:**

The findings signify the unique sociocultural context in Hong Kong, which is deeply affected by Chinese traditions, Western individualism, and neoliberal ideals. Overall, the study provides robust support for the CS as a reliable and valid measure for cross-cultural research on compassion and yields evidence-based implications for compassion interventions.

## Introduction

The concept of compassion generally refers to an altruistic desire to care for others who are experiencing pain, distress, or hardship (Neff, 2003; Pommier et al., 2020). Compassion is found to reduce stress levels, enhance emotional regulation, and contribute to better mental health outcomes (Yela et al., 2022). Moreover, it helps build interpersonal relationships, promotes prosocial behavior within cogenemmunities, and fosters social cohesion at large (Kirkland et al., 2023). For adolescents, compassion is crucial as they navigate the myriad developmental tasks inherent in transitioning from childhood to adulthood, including forming self-identity, seeking autonomy and independence, establishing peer relationships, and exploring educational/vocational pathways (Bluth et al., 2018; Branje et al., 2021; Cunha et al., 2016; Melendro et al., 2020). Compassionate adolescents tend to exhibit the ability to understand others’ viewpoints, which enhances the quality of social interactions and fosters more profound connections with peers and family members (Breines et al., 2015). On the contrary, a lack of compassion among adolescents may jeopardize their mental health and hinder their social relationships, ultimately impeding their overall development. Promisingly, research indicates that compassion is a malleable trait that can be cultivated through various interventions, such as mindfulness-based programs and compassion-focused therapy (Yela et al., 2022). By equipping adolescents with the skills to be more compassionate toward others, these interventions can help buffer against the adverse effects of stressors and generate a desire to help (Cheang et al., 2019).

Measuring compassion with a reliable and valid instrument is thus essential. It is noteworthy, however, that cultural norms and values may influence the expression and interpretation of compassion. Cultures influenced by Eastern philosophies, such as Confucianism, may have a stronger emphasis on compassion as a core component of well-being and spiritual growth, while Western culture may be more closely associated with individualistic values of self-care and personal fulfillment (Neff et al., 2008). Due to its colonial past, Hong Kong was under British rule for nearly 150 years before being returned to China in 1997 as a special administrative region. On the one hand, it is a highly industrialized metropolitan city heavily influenced by Western cultures and neoliberal ideals, such as the pursuit of personal achievement and self-reliance. On the other hand, Hong Kong has deep roots in traditional Chinese culture, represented in its predominantly (95%) Chinese population, which emphasizes the importance of family bonding and social harmony (Lin and Ho, 2009; Ngai et al., 2018). The unique cultural context, which blends Confucian values of social harmony with Western individualism, creates both opportunities and challenges for fostering compassion. Hong Kong adolescents growing up in this unique sociocultural environment may yearn for great independence and autonomy, yet as influenced by Confucianism, they may also value interdependence that stresses harmonious interpersonal connections (Neff et al., 2008; Ngai et al., 2018). As such, Hong Kong presents a compelling case for evidence-based compassion research. Given the city’s distinct cultural norms and values, it would be intriguing to explore how these factors shape the expression and interpretation of compassion among adolescents. Examining the applicability of compassion measurement instruments developed and validated in Western contexts could also yield valuable insights into the nuances of compassion within Hong Kong’s unique cultural landscape. Additionally, understanding these dynamics could inform culturally-attuned approaches to fostering compassion and social well-being among adolescents in Hong Kong and similar cultural contexts.

### Instruments to measure compassion

Compassion is described as a mental state and an active effort to alleviate the suffering of others (Strauss et al., 2016). This conceptualization of compassion toward others is notably distinct from the notion of self-compassion or compassion for oneself, as it encompasses the emotional response that arises when bearing witness to another individual’s suffering, as well as the subsequent desire to provide aid and assistance (López et al., 2018). In a comprehensive systematic review, Strauss et al. (2016) identified five key components that constitute compassion directed toward the suffering of others. These include: (1) recognition of suffering; (2) understanding its universality; (3) concern for those who are suffering; (4) tolerating the distress associated with the witnessing of suffering; and (5) motivation to act or acting to alleviate the suffering. As such, compassion toward others is more closely aligned with other-focused attitudes and dispositions, and entails less of the uncompassionate responses, alongside greater compassionate responses in terms of emotional reactions, cognitive understanding, and attentional focus on the suffering of others (Pommier et al., 2020). Notably, adolescence is a critical developmental stage characterized by cognitive, physiological, and neurological changes. The pursuit of self-identity, autonomy, and peer acceptance during adolescence can lead to elevated stress, negative self-perception, and greater susceptibility to emotional distress (Sousa et al., 2022). The Hong Kong education system places a strong emphasis on academic achievement, fostering a highly competitive environment for adolescents. Given that intense focus on academic performance can cause significant stress and anxiety among students, compassion components such as “recognizing suffering” and “acting to alleviate the suffering” are particularly relevant in this context. These components help assess adolescents’ ability to identify emotional struggles in themselves and others and respond appropriately (Neff and McGehee, 2010).

Due to the differences in population and context, investigators in separate geographical areas have validated compassion measurement tools in a variety of ways. For example, the Scale of Compassion for Others was a subscale of the Compassion Engagement and Action Scale (CEAS) developed by Gilbert et al. (2017). It was designed to measure the sensitivity to the suffering of others and how the individual is motivated to prevent or alleviate the suffering of others. In the past few years, this scale has been validated among adults and adolescents in many different countries around the world (including adult samples from the UK, Portugal, and the USA and adolescent samples from Sweden and Portugal), and both the total scale and the subscales all showed good validity and reliability (Asano et al., 2020; Cunha et al., 2023; Gilbert et al., 2017; Henje et al., 2020). Besides the CEAS, other compassion scales have also been developed. For instance, the Compassion Scale-20 (Nas and Sak, 2021), the Relational Compassion Scale (Hacker, 2008), the Sussex-Oxford Compassion for Others Scale (Gu et al., 2020), and the Santa Clara Brief Compassion Scale (Plante, 2022) are also useful measurement tools that can measure compassion for others.

Despite the wide use of these scales, some limitations still exist. Specifically, most of them are single-dimensional scales or only a subscale of the compassion scale. Furthermore, they also lack exploration from the perspective of theoretical and cultural differences. Hence, based on this, Pommier et al. (2020) developed a new Compassion Scale (CS), which uses a similar operational definition of the Self-Compassion Scale (Neff, 2003) to measure compassion for others. The CS comprises four subscales: kindness, common humanity, mindfulness, and indifference. Compared with the previous compassion scales, the CS of Pommier et al. (2020) is multidimensional and better meets the five criteria proposed by Strauss et al. (2016) (see p. 3, para. 2). Kindness items involve component 3: concern for those who are suffering, and component 5: the motivation to alleviate the suffering. Mindfulness items encompass component 1: recognition of suffering, and reverse-coded indifference items comprise component 4: tolerating the distress associated with the witnessing of suffering. Common humanity items tap into component 2: understanding the universality of suffering (Pommier et al., 2020). As such, the CS is more comprehensive than other commonly used compassion measures by including the dimension of common humanity, which is missing in existing compassion measures (Pommier et al., 2020). Additionally, it has demonstrated stronger psychometric properties through the validation of its factor structure across diverse adult samples in six separate studies (Pommier et al., 2020). In recent years, the CS has been validated among Italian and Swedish adults (Lucarini et al., 2023; Wolgast et al., 2024) with the original factor structure. Sousa et al. (2022) also investigated the psychometric properties of the CS in community adolescents and adolescents with behavioral disorders in Portugal, yielding the same construct components. Still, research assessing the applicability of the CS in an Eastern Asian context is scarce.

### Relationships between compassion with social connectedness and self-efficacy

For adolescents, it is critical to recognize compassion’s relation with social connectedness and self-efficacy, which have implications for their social well-being and mental health (Kleppang et al., 2023). Existing research shows reciprocity between compassion and social connectedness (Seppälä et al., 2017). When individuals exhibit compassion, they tend to form stronger bonds with others, which help generate positive social emotions and increase prosocial behavior, enhancing social connectedness (Luengo Kanacri et al., 2021). On the other hand, close relationships and social networks offer opportunities for individuals to express and receive compassion. This reciprocity strengthens interpersonal bonds and contributes to well-being (Seppälä et al., 2017). Likewise, research has shown that individuals who regularly engage in compassionate behaviors tend to have higher levels of self-efficacy (Neff and Pommier, 2013). According to social cognitive theory, self-efficacy refers to an individual’s belief in their capacity to organize and execute courses of action required to attain designated types of performances (Bandura, 2023). Engaging in compassionate actions provides opportunities for mastery experiences. When individuals successfully help others or show kindness in various situations, they gain a sense of competence and belief in their ability to handle similar situations in the future (Kleppang et al., 2023). Strauss et al. (2016) explored the role of compassion-focused interventions in enhancing self-efficacy among adolescents. Their findings suggested that interventions promoting compassion not only improved adolescents’ social relationships but also boosted their self-beliefs in managing challenges in life. Given the above, we hypothesize that compassion positively correlates with social connectedness and self-efficacy.

### Gender and age differences in compassion

Studying compassion among adolescents is a matter of great importance as adolescence lays the foundation for their ultimate development into socially responsible adults. Compassion not only shapes adolescents’ interpersonal relationships but also influences their contributions to society, making it a pivotal aspect of their growth and well-being (Bluth et al., 2018; Cunha et al., 2016). A substantial body of literature has documented compassion and its relation to gender and age during adolescence. Studies consistently show that adolescent girls tend to report higher levels of compassionate concern and engage more frequently in compassionate behaviors than boys (Andrews et al., 2021; Bluth et al., 2017; Chaplin and Aldao, 2013; Löffler and Greitemeyer, 2023). In a recent study conducted among Portuguese adolescents, girls living in the community presented higher levels of compassion toward others than did boys (Sousa et al., 2022). Similarly, women generally scored higher than men in compassion among a sample of Swedish adults (Wolgast et al., 2024). However, compassion and its variation with age during adolescence remain inconclusive. Eisenberg’s developmental model posits that adolescents undergo significant neurological, cognitive, and socioemotional growth processes, contributing to an increased understanding of others’ emotions and perspectives, facilitating greater compassion as adolescents mature (Luengo Kanacri et al., 2021). In contrast, others argue that younger adolescents, due to the heightened importance of peer relationships, may exhibit a greater tendency to seek social acceptance and, hence, a stronger motivation to alleviate others’ suffering during this developmental stage (Marston et al., 2010). In the Hong Kong context, it remains to be seen how compassion varies with gender and age among adolescents.

### The present study

Although the previous studies (Lucarini et al., 2023; Pommier et al., 2020; Wolgast et al., 2024) have provided meaningful results to support the validity and reliability of the 16-item CS, the validation studies were primarily conducted in Western contexts. The unique cultural blend of East and West in Hong Kong, influenced by Confucianism, neoliberalism, and individualism, presents an interesting case for studying compassion and seeing how these cultural forces interact regarding compassion. In addition, current studies validating the CS primarily focus on adults, whose psychological characteristics differ from those of adolescents in terms of compassion. Thus, validating a reliable, culturally sensitive instrument to measure compassion among adolescents is essential. First, we sought to verify the factor structure and psychometric properties of the CS among adolescents in Hong Kong. Then, we assessed convergent and discriminant validity. Furthermore, we examined how compassion and its subscales varied with gender and age from a cross-cultural perspective. Additionally, we assessed the concurrent validity of the CS by analyzing the correlations between the total scale and its subscales with social connectedness and self-efficacy. These analyses collectively ensured the scale’s cross-cultural robustness and appropriateness among adolescents in the Hong Kong context.

## Methods

### Participants and procedure

This study employed a stratified random sampling approach to recruit a representative sample of secondary school students using geographical regions as the stratifying factor. Written invitations and consent forms were sent to 11 secondary schools across different regions in Hong Kong to invite students to participate in the study. Participation was voluntary, and confidentiality was assured in the written invitations and consent forms prior to the recruitment process. The invited students then completed a self-administered questionnaire anonymously. In total, 1,193 secondary school students in grades 7 to 11 completed the questionnaires. The study obtained ethical approval from the ethics review committee of a major university in Hong Kong.

### Measures

*The Compassion Scale* was developed by Pommier et al. (2020) to measure the degree of one’s compassion in terms of emotional response, cognitive understanding, and attention to suffering. The CS consisted of 16 items that fell into four subscales: kindness, common humanity, mindfulness, and indifference. Participants were asked to indicate how often they felt or behaved in a stated manner on a 5-point Likert scale from “1 = *Rarely*” to “5 = *Almost always*.” In calculating the overall compassion level, the subscale of indifference was reverse-coded. A grand mean of all items suggested a total compassion score, with a higher score indicating a higher level of compassion. The total scale had a Cronbach’s alpha value of 0.904, indicating satisfactory internal consistency. The CS was translated into Chinese and verified through back-translation procedures with its semantic equivalence of the Chinese version to the original scale. The Chinese version of the CS was applied in this study.

The Social Connectedness Scale is an adaptation of Kern et al.’s (2015) well-being measurement scale. It has been culturally adapted and validated in the Hong Kong context (Ngai et al., 2021) and was used to measure one of the well-being outcomes among young patients with chronic illnesses in Hong Kong, demonstrating high reliability in that study. This study adopted Ngai et al.’s adapted version, which encompasses five items to assess participants’ perceived quality and satisfaction with their social relationships (Ngai et al., 2021). A sample statement would be, “My relationships are supportive and rewarding.” Participants indicated how often certain situations had happened to them by rating them on a 5-point Likert scale, ranging from “1 = *Never*” to “5 = *Always*.” This scale had satisfactory internal reliability with the value of Cronbach’s alpha at 0.921.

The General Self-Efficacy Scale (GSE), developed by Schwarzer and Jerusalem (1995), consists of ten items that measure an individual’s belief in their ability to cope with stressful or challenging situations. The GSE has been translated into multiple languages and validated across diverse cultural and societal contexts (Schwarzer et al., 1997). The Chinese version of the scale has been validated among undergraduates and adolescents, demonstrating strong reliability and cultural appropriateness (Schwarzer and Jerusalem, 1995; Zeng et al., 2022). Participants were asked to respond to statements on a 4-point Likert scale ranging from “1 = *Not at all true*” to “4 = *Exactly true*.” A sample statement would be, “I can always manage to solve difficult problems if I try hard enough.” The mean score was calculated by averaging the sum of all items, with a higher score indicating higher levels of self-efficacy. This scale had satisfactory internal reliability, with Cronbach’s alpha value at 0.944.

### Data analysis

First, descriptive analyses were compiled to describe the sociodemographic characteristics of the participants. Then, the sample was randomly divided into two subsamples (Subsample 1, *N* = 597; Subsample 2, *N* = 596) to conduct exploratory and confirmatory factor analyses, respectively. The Kaiser–Meyer–Olkin (KMO) measure of sampling adequacy and Bartlett’s test of sphericity were applied before factor extraction to ensure the data fitness for exploratory factor analysis (EFA). Next, we performed EFA for the scale and calculated item-total correlations of Cronbach’s alpha internal consistency coefficients. In this step, iterated principal factor analysis was applied in the current study, and we selected the criteria proposed by Comrey and Lee (2013) to assess item loadings: 0.32 poor, 0.45 fair, 0.55 good, 0.63 very good, and 0.71 excellent.

Next, confirmatory factor analysis (CFA) was used to validate the factorial model obtained from EFA using the other random half of the sample (Subsample 2, *N* = 596). We adopted the model fit criteria proposed by Hu and Bentler (1999) and Sathyanarayana and Mohanasundaram (2024). Indices such as the Chi-square statistic (χ^2^) and related degrees of freedom (*df*), comparative fit index (CFI), root mean square error of approximation (RMSEA), and standardized root mean square residual (SRMR) were used to assess the goodness of fit of the model in this step. CFA was used further to examine the factorial structure of the CS. Goodness-of-fit indices for an acceptable model fit were as follows: CFI ≥ 0.90, RMSEA ≤0.08, and SRMR ≤0.08, with a 90% confidence interval (CI).

Subsequently, the constructs’ convergent validity and discriminant validity of the constructs were examined using a series of statistical methods. Convergent validity is used to assess whether items that are related are actually observed to be related in the data. We evaluated convergent validity using three key indicators: standardized factor loadings, Average Variance Extracted (AVE), and Composite Reliability (CR). Factor loadings should exceed 0.50, indicating that the items account for a substantial portion of the variance in their respective construct (Hair, 2010). The AVE, which represents the average amount of variance a construct explains in its items, should be greater than 0.50, suggesting that the construct accounts for more than 50% of the variance in its indicators (Fornell and Larcker, 1981; Hair, 2010). The CR, which is used to assess the internal consistency of items measuring a given construct, should exceed 0.70, indicating good construct reliability (Hair, 2010).

Discriminant validity is used to assess whether constructs that are theoretically distinct are also empirically distinct. We examined discriminant validity using several established methods. First, using Cheung et al.’s (2024) approach, we calculated the 95% confidence intervals (CIs) of correlations between construct pairs. If the CI excludes 1.0, this suggests an extremely low possibility of a perfect correlation between the two factors, supporting discriminant validity. Then, we compared model fit between an unconstrained model (in which constructs are separate) and a constrained model (where two constructs are combined into one) using the Akaike Information Criterion (AIC) and Bayesian Information Criterion (BIC). Lower AI or BIC values indicate a better model fit. A significantly better fit (*p* < 0.05) for the unconstrained model provides evidence that the constructs are distinct and should be treated as separate.

Multigroup CFA analysis was further performed to further assess the model fit of the CS across subgroups of gender (male/female), age (11–13/14–18 years), school type (Chinese/non-Chinese), and grade (grades 7–9/ grades 10–11), respectively. After that, an independent sample *t*-test was used to examine the score differences on the CS and its subscales categorized by gender and age subgroups.

Concurrent validity, which refers to the extent to which a new measure correlates with previously validated measures of related constructs (DeVellis and Thorpe, 2021; Furr, 2021), is typically assessed through correlational analyses between the new scale and established measures. As reviewed in the Introduction, compassion and social connectedness share a reciprocal relationship (Seppälä et al., 2017). Compassionate individuals tend to form stronger bonds with others through positive social emotions and increased prosocial behaviors, while close relationships and social networks, in turn, provide opportunities to express and receive compassion (Luengo Kanacri et al., 2021). In addition, existing research indicates that compassionate individuals exhibit higher levels of self-efficacy as they develop a sense of competence and confidence in their ability when successfully helping others (Kleppang et al., 2023). To verify the concurrent validity of the Compassion Scale, we examined its relationship with two theoretically related constructs: social connectedness and general self-efficacy, while controlling for age, gender, ethnicity, grade, and school type in the analysis. A correlation coefficient was considered weak when the absolute value of *r* was less than 0.30, moderate when *r* ranged from 0.30 to 0.50, and strong when *r* exceeded 0.50 (Cohen, 1992; Kim, 2015). Data analysis was conducted using Stata version 17.0.

## Results

### Profile of participants

Of the total 1,193 participants, 517 were females (43.34%). A dominant majority (91.70%) were of Chinese ethnicity, and three-quarters (75.36%) were from grades 7–9. Participants had a mean age of 13.80 years, ranging from 11 to 18 (see Table 1 for details). The total sample was randomly separated into two subsamples to conduct the exploratory factor analysis and confirmatory factor analysis.

**Table 1:** Demographic characteristics of the sample.

		Percent	*N*
Gender	Female	43.34	517
	Male	56.66	676
Ethnicity	Chinese	91.70	1,094
	Non-Chinese	8.30	99
School Type	Chinese School	68.15	813
	Non-Chinese School	31.85	380
Grade	7–9	75.36	899
	10–11	24.64	294
Age	Mean (*SD*)	Min	Max
	13.80 (1.41)	11	18

### Exploratory factor analysis (EFA)

In this study, the examination of the correlation matrices revealed the presence of all factor loading above 0.5 (0.505 to 0.862). The KMO analysis yielded an index of 0.923, and Bartlett’s test of sphericity was significant (χ^2^ = 44785.715, *df* = 120, *p* < 0.001), indicating that the data set was satisfactory to perform factor analysis (see Table 2 for details). The scree plot identified three factors: factor 1 (benevolence, the renamed factor combining mindfulness and kindness), factor 2 (common humanity), and factor 3 (indifference). They contributed 83.06% of the common variance, with individual factors contributing 44.24, 15.15, and 23.68%, respectively.

**Table 2:** Rotated factor loadings matrix from EFA (*N* = 597).

Items		Factor 1 *Benevolence*	Factor 2 *Common Humanity*	Factor 3 *Indifference*
M1	I pay careful attention when other people talk to me about their troubles.	0.810		
M2	I notice when people are upset, even if they do not say anything.	0.625		
M3	I listen patiently when people tell me their problems.	0.678		
M4	When people tell me about their problems, I try to keep a balanced perspective on the situation.	0.659		
K1	If I see someone going through a difficult time, I try to be caring toward that person.	0.862		
K2	I like to be there for others in times of difficulty.	0.736		
K3	My heart goes out to people who are unhappy.	0.525		
K4	When others feel sadness, I try to comfort them.	0.714		
CH1	I realize everyone feels down sometimes; it is part of being human.		0.589	
CH2	I feel it’s important to recognize that all people have weaknesses and no one’s perfect.		0.511	
CH3	I feel that suffering is just a part of the common human experience.		0.505	
CH4	Despite my differences with others, I know that everyone feels pain just like me.		0.574	
I1	I am unconcerned with other people’s problems.			0.718
I2	I think little about the concerns of others.			0.760
I3	I try to avoid people who are experiencing a lot of pain.			0.746
I4	I cannot really connect with other people when they are suffering.			0.731

### Item and reliability analysis

Internal consistency was estimated for the CS using Cronbach’s coefficient. The values were 0.904 for the total scale and 0.904, 0.769, and 0.830 for the benevolence, common humanity, and indifference subscales, respectively. In terms of item-total correlations, all items revealed moderate to strong item-total correlations ranging from 0.410 (Item I1 of the indifference subscale) to 0.765 (Item M4 of the benevolence subscale). These results indicate that the internal consistency of the three subscales was strong, and the overall CS had good reliability (see Appendix I for details).

### Confirmatory factor analysis (CFA)

**Figure 1:**
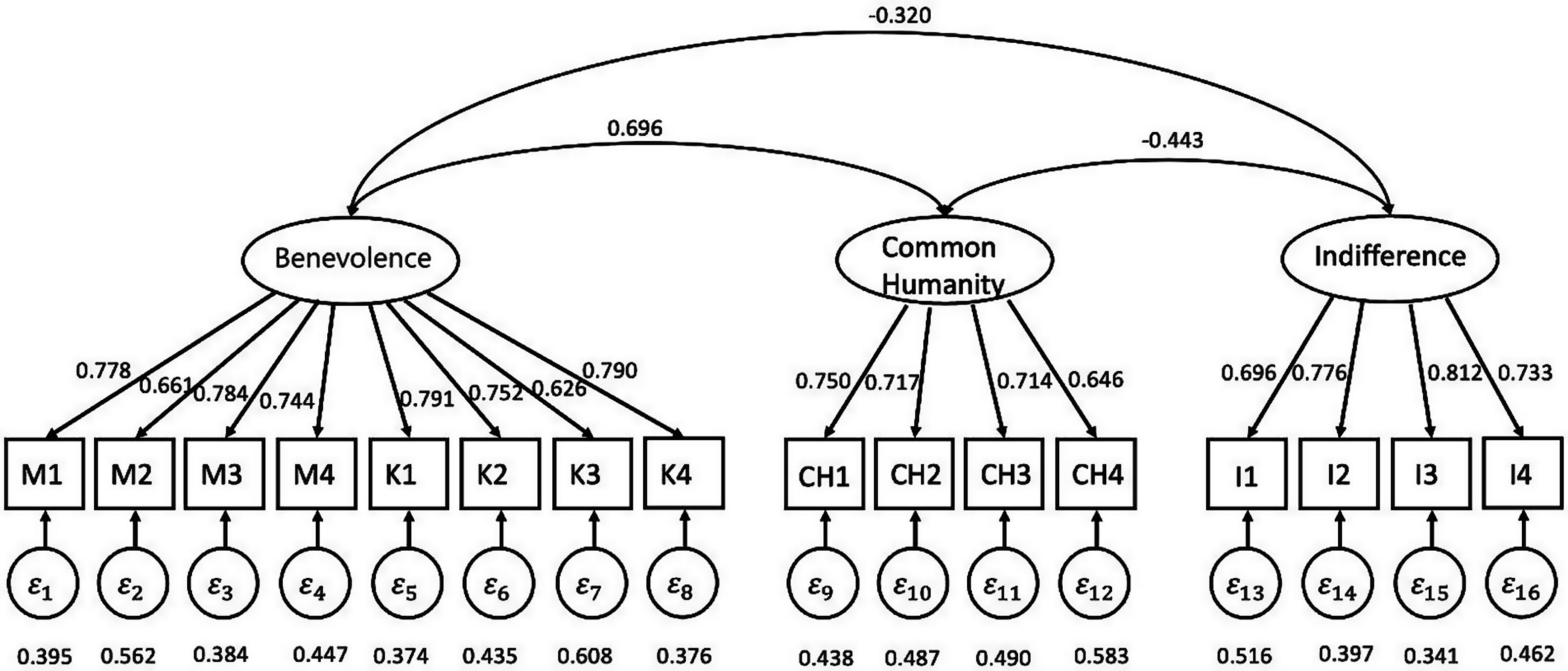
Validation of the factor structure with CFA (*N* = 596).

CFA was conducted to validate the factorial structure of the CS in Subsample 2. Maximum likelihood estimation was used to estimate the model. Results revealed an adequate fit of the hypothesized model to the data from Subsample 2, χ^2^ (101) = 474.389, *p* < 0.001, CFI = 0.926, RMSEA = 0.079, SRMR = 0.063. The CFA results showed acceptable model fit indices. Regarding item factor loadings, the three-factor correlated CFA model was well-defined. All factor loadings were statistically significant (*p* < 0.001) and ranged from 0.626 to 0.812 (see Figure 1 for details).

### Convergent and discriminant validity

Incorporating the Fornell-Larcker AVE criterion for convergent validity along with construct reliability, Hair et al. (2019) suggested that there was evidence for convergent validity when all three of the following conditions are fulfilled: (a) CR values are 0.7 or greater, (b) all standardized factor loadings *λ* are 0.5 or greater, and (c) AVE values are 0.5 or greater. Our findings fulfill multiple criteria when examining convergent validity (Hair et al., 2019). The convergent validity analysis through the AVE was also good for all subscales, suggesting that its observed variables well explain the latent factors (AVE benevolence = 0.589, AVE common humanity = 0.514, AVE indifference = 0.568). All CR values of the three subscales were also above 0.700. These indicated that each construct was relatively compact without being confused with other constructs (see Appendix II for details).

As expected, the 95% CI of the correlations between pairwise factors (benevolence and common humanity; benevolence and indifference; common humanity and indifference) does not contain one in Subsample 2 and the total sample, suggesting they are measuring distinct constructs (see Appendix III for details). In addition, the correlations are lower than the 0.75 threshold (Voorhees et al., 2016), which also serves as strong evidence for discriminant validity. We calculated the likelihood ratio test results of the unconstrained model (the current model) versus the constrained model formed by combining benevolence and common humanity into one factor (as they have the highest correlation, 0.696 in sub-sample 2 and 0.675 in the total sample) with model fit statistics. In both the total sample and Subsample 2, the constrained models have higher AIC and BIC values than the unconstrained models, suggesting that the unconstrained models better fit the data. Additionally, the χ^2^ tests indicate that the unconstrained models significantly improve model fit compared to the constrained models, as indicated by the *p*-values less than 0.001 (see Appendix IV for details). These results suggest that the current model better fits the data in both the total sample and Subsample 2. Overall, the above results show that the scale has good convergent and discriminant validity.

### Multigroup CFA analysis

We further tested the multigroup analysis by gender (male and female), age (11–13 and 14–18 years), grade (grades 7–9 and grades 10–11), and school type (Chinese and non-Chinese). As presented in Table 3, nearly all subgroups obtained satisfactory model fit index in CFI, RMSEA, and SRMR. The female and grades 10–11 subgroups show marginal model fit in CFI and RMSEA. The results indicate the structural validity across various adolescent groups in Hong Kong.

**Table 3:** The goodness of fit for the multi-group CFA models.

	Full Sample	Gender		Age		Grade		School Type	
		Male	Female	Younger	Older	7–9	10–11	Chinese	Non-Chinese
*N*	1,193	676	517	599	594	899	294	813	380
Chi-square	812.587	532.222	477.504	486.625	479.731	579.641	430.374	743.738	316.241
Degree of freedom	101	101	101	101	101	101	101	101	101
*P*-value	<0.001	<0.001	<0.001	<0.001	<0.001	<0.001	<0.001	<0.001	<0.001
CFI	0.927	0.929	0.899	0.919	0.924	0.932	0.883	0.911	0.915
RMSEA 90% CI	0.077 (0.072, 0.082)	0.080 (0.073, 0.086)	0.085 (0.077, 0.093)	0.080 (0.073, 0.087)	0.080 (0.072, 0.087)	0.073 (0.067, 0.078)	0.095 (0.075, 0.105)	0.089 (0.083, 0.095)	0.075 (0.066, 0.084)
SRMR	0.062	0.057	0.071	0.070	0.058	0.061	0.064	0.065	0.065

### Gender and age differences

As shown in Table 4, we used an independent sample *t*-test to present the mean differences of the CS and subscales by gender and age. No significant gender differences in the overall compassion score or the benevolence and common humanity subscales were observed (*p* > 0.05). In the indifference subscale, girls gained significantly lower scores than boys (*M* = 2.702 vs. *M* = 2.933, *p* < 0.001), suggesting that, on average, girls showed a higher level of compassionate concern for others than boys. On the other hand, younger adolescents were found to be more compassionate than their older counterparts (*M* = 3.504 vs. *M* = 3.414, *p* < 0.001). Specifically, younger adolescents scored significantly higher in the benevolence subscale than older ones (*M* = 3.626 vs. *M* = 3.506, *p* < 0.001), while no significant age differences were observed in the other subscales (*p* > 0.05).

**Table 4:** Mean comparisons of the CS and its subscales by gender and age and partial correlations of compassion with Social connectedness and general Self-efficacy.

	Gender				Age			
	Mean		T Test		Mean		T Test	
	Female	Male	*diff*	*p*	Younger	Older	*diff*	*p*
Compassion^#^	3.480	3.444	0.037	0.267	3.504	3.414	0.089	**0.007**
Benevolence	3.564	3.568	−0.003	0.941	3.626	3.506	0.119	**0.013**
Common Humanity	3.487	3.570	−0.083	0.099	3.571	3.497	0.075	0.135
Indifference	2.702	2.933	−0.237	**<0.001**	2.807	2.859	−0.041	0.473

	Social Connectedness	General Self-Efficacy
Compassion^#^	0.589^***^	0.500^***^
Benevolence	0.588^***^	0.463^***^
Common Humanity	0.482^***^	0.391^***^
Indifference	−0.258^***^	−0.383^***^

^#^Four items of indifference were reversely coded in calculating the total compassion score. Age, gender, ethnicity, grade, and school type were controlled in partial correlation analyses. ^***^p < 0.001.

### Concurrent validity

Concurrent validity was examined through the correlations between the total scale and subscales of the CS with social connectedness and self-efficacy. As displayed in Table 4, the CS was significantly correlated with measures of social connectedness (*r* = 0.589, *p* < 0.001) and self-efficacy (*r* = 0.500, *p* < 0.001). Apart from the total scale, the three subscales of benevolence, common humanity, and indifference were significantly (*p* < 0.001) correlated with social connectedness and self-efficacy, suggesting sufficient concurrent validity.

## Discussion

The present study supports the applicability of the CS among adolescents in Hong Kong. We extracted three factors from EFA results: benevolence (eight items), common humanity (four items), and indifference (four items). Factor loadings on each latent sub-construct ranged from 0.505 to 0.862. Thus, no item needs to be removed. The subsequent CFA confirmed the three-factor structure, and multigroup CFA revealed its goodness-of-fit model across gender, age, grade, and school type. Furthermore, the convergent validity of the CS was supported by high AVE values (all above 0.50) and strong CR values (all above 0.70), indicating that the items within each construct consistently measure the same underlying concept. Discriminant validity was established through model comparisons, which confirmed that the constructs were empirically distinct. The concurrent validity of the CS was also supported by significant correlations between the total scale and its subscales with social connectedness and general self-efficacy. These results collectively demonstrate that the Chinese version of the CS has strong psychometric properties, making it a reliable and valid tool for assessing compassion among adolescents in Hong Kong.

In this study, factor correlations were all statistically significant, indicating that the three factors are related elements of an overall compassion construct. Furthermore, the correlation between the subscales is consistent with the previous Western research findings (Lucarini et al., 2023; Pommier et al., 2020; Wolgast et al., 2024). For example, the mindfulness subscale and kindness subscale exhibited the highest correlation, while relatively weak correlations were observed between common humanity and indifference in the current study. It is similar to the findings reported in recent articles, which validate the Swedish and Italian versions of the CS (Lucarini et al., 2023; Wolgast et al., 2024). Specifically, in all these studies, common humanity and indifference exist as two distinct dimensions of compassion. Due to the pervasive Western influence in the colonial period, Hong Kong has developed a culture that heavily emphasizes individualism and self-sufficiency, in contrast to the more collectivist orientation typical of many East Asian societies (Ngai et al., 2018). As a result of this cultural orientation toward individualism, the definitions and interpretations of the concepts of common humanity and indifference have also diverged in this context. Common humanity is understood as a common human experience of coming across hardships and having a sense of connection to those suffering. Differently, indifference evolved from uncompassionate attitudes toward others. It represents the uncompassionate attitudes toward others and is opposite to common humanity (Pommier et al., 2020). Hence, just as in the original version of the scale and the Western findings, common humanity and indifference were still two independent factors in the current study.

In our findings, the strong connection between kindness and mindfulness is in accordance with the original CS (Pommier et al., 2020) and the Swedish and Italian studies. The major difference is that, despite their strong correlation, these two concepts were regarded as two separate dimensions in previous research while it becomes one dimension in our study. In Western cultures, kindness has been characterized as exhibiting care and concern for others who are suffering, coupled with the motivation to provide support to those in need (Pommier et al., 2020), and mindfulness usually refers to a balanced awareness that shows the willingness to pay attention and listen to others when they are suffering while not becoming lost in the pain of others (Wolgast et al., 2024). Thus, the two concepts are interrelated under compassion yet as independent dimensions.

In comparison, the notions of kindness and mindfulness find their roots in traditional Chinese culture in Hong Kong. According to Confucianism, kindness (in Chinese *Ci*慈) often involves an other-regarding orientation and a tender loving concern for others (Li, 2023). Unlike in Western contexts, Confucian teachings emphasize kindness not only as a virtue for individuals but also as a norm to maintain social harmony, reflecting the interconnectedness of individuals and their responsibilities toward others in society. Individuals are encouraged to prioritize the well-being of others alongside their own interests, contributing to a harmonious and ethical social order (Lin and Ho, 2009). On the other hand, mindfulness (in Chinese *Jing*静) in Confucianism refers to respectful attention or self-cultivation, which involves being mindful of one’s duties, responsibilities, and ethical principles to achieve goodness in oneself (Tan, 2019; Tan, 2021). Similar to kindness under Confucianism, mindfulness focuses on continuous self-awareness and improvement to align one’s behavior with moral principles and societal expectations (Tan, 2019). That is, kindness and mindfulness from the Confucian perspective both encompass a genuine concern for others, involve a desire to alleviate suffering, and ultimately relate oneself to others in society. In our sample, the two highly correlated dimensions of kindness and mindfulness merged as one factor, which we coined “benevolence” (in Chinese *Ren*仁), a key concept in Confucian ethics (Li, 2023) that denotes a caring concern for others to develop a virtuous character for harmonious relationships. It regards kindness and mindfulness as fundamentally interpersonal, whereas Western cultures generally view mindfulness as an internal and cognitive process (Cheang et al., 2019). Thus, with a combined factor of benevolence, together with common humanity and indifference, our sample represents a unique cultural application of the CS under the influence of Confucianism in Hong Kong.

Regarding gender differences, the findings of group comparisons suggest that girls, on average, showed a dramatically higher compassionate concern toward others than boys (*p* < 0.001), as indicated in the indifference subscale. This gender difference in compassionate concern can be attributed to various factors, such as gender socialization and societal expectations (Andrews et al., 2021). From early childhood, girls are often socialized to be nurturing and caring toward others, which stresses the emotional and affective aspects of compassion (Andrews et al., 2021). The importance of interpersonal relationships and emotional support is emphasized during girls’ socialization process, fostering a greater inclination toward compassionate concern for others’ problems. Additionally, girls may be encouraged or expected to prioritize relationships and demonstrate compassionate concern as part of their feminine identity (Andrews et al., 2021). On the contrary, boys are commonly socialized with emotional restrictiveness patterns, which may restrain their expression of compassionate concern toward others (Sousa et al., 2022). In general, Hong Kong adolescents show gender differences in the affective aspect of compassion, similar to their Western counterparts. For example, the studies among Italian and Swedish samples show that women gain higher scores in the CS total and subscales (Lucarini et al., 2023; Wolgast et al., 2024).

Interestingly, compassion demonstrated a slight tendency to increase with age in the community sample observed by Pommier et al. (2020), while in our sample, older adolescents reported lower overall compassion scores than their younger counterparts. While cognitive and emotional development during adolescence may contribute to an increased understanding of others’ emotions and perspectives, older adolescents may experience emotional exhaustion due to heavy academic stress that threatens their mental health and inhibits their inclination to care for others (Chen et al., 2022). For example, an experiment shows that youth tend to ignore others’ needs in a competitive environment (Kirkland et al., 2023). Similarly, an ethnographic study demonstrates that the more severe the competition is, the less compassion the youth show (Zhao, 2015). Moreover, the overall compassion score in Hong Kong is lower than those observed in Western samples (Lucarini et al., 2023; Pommier et al., 2020; Sousa et al., 2022). For example, the mean CS score for Hong Kong adolescents is 3.31, whereas it is 3.93 in the Portuguese adolescent sample (Sousa et al., 2022). Confucian norms generally discourage overt emotional expression, which can suppress outward displays of compassion (e.g., verbal reassurance, physical comfort), whereas Western cultures emphasize self-expression, emotional openness, and personal altruism—key aspects of most compassion metrics (Kim et al., 2008; Strauss et al., 2016). Also, under the neoliberal society emphasizing competition, Hong Kong’s education system is renowned for its rigor and competitiveness, where students face immense pressure to excel academically (Chyu and Chen, 2022). The demanding nature of academic commitments is even manifested when students enter senior grades, leaving older adolescents with limited time for social interactions and compassionate acts. Another possible explanation could be the influence of neoliberal social norms that promote individual responsibility and self-reliance in Hong Kong. As they grow, Hong Kong adolescents may prioritize personal achievement and success, sometimes at the expense of compassion toward others (La Londe and Verger, 2022). Immersed in a competitive environment, Hong Kong adolescents are likely to perceive others as competitors rather than as individuals deserving of compassion. This dynamic can affect interpersonal relationships and the development of prosocial behaviors, which makes older adolescents less compassionate than younger ones in Hong Kong. Though perspective-taking, prosocial responding, and moral reasoning, the capabilities that are closely related to compassion, are found to increase with age in Western context (Kunzmann et al., 2018; Luengo Kanacri et al., 2021), an increase in age does not necessarily lead to an increase in compassion in Hong Kong and the overall compassion score among adolescents is relatively lower given its special sociocultural context.

In addition, our study established concurrent validity by showing significant and positive correlations between compassion and its subscales with social connectedness and self-efficacy (*p* < 0.001). These findings reveal important insights into interpersonal relationships and adolescent well-being. For adolescents, compassion and prosocial behaviors contribute to building and maintaining social support networks, which, in turn, enhance social connectedness and provide resources for coping with stress and adversity (Förster and Kanske, 2022; Seppala et al., 2013). Likewise, engaging in compassionate acts can provide adolescents with a sense of mastery over their environment and relationships, enhance their self-perception, and reinforce their sense of efficacy in social interactions (Kleppang et al., 2023).

## Implications

The empirical findings in our study provide fruitful implications for compassion research and interventions in Hong Kong. Validating a compassion scale in Hong Kong allows researchers to examine how cultural values and norms influence adolescents’ understanding and expression of compassion, considering Hong Kong’s unique blend of Chinese traditions, Western influences, and neoliberal ideals that shape adolescents’ social interactions and moral reasoning (La Londe and Verger, 2022). In addition, it contributes to cross-cultural research by comparing compassion levels and correlates across different societies, which can identify universal aspects of compassion as well as cultural variations in its expression and development, informing theories of moral development and cultural psychology. In practice, nongovernmental organizations and schools are able to design compassion programs for the well-being of local adolescents with a valid and culturally compatible tool to measure compassion. For example, social–emotional learning frameworks that emphasize interpersonal skills and moral development can be integrated into mindfulness-based interventions for senior students (Cheang et al., 2019), which can not only promote compassion among adolescents but also help cultivate a society caring for one another in Hong Kong. Additionally, our study establishes a regionwide standard to measure compassion via the CS and its benevolence, common humanity, and indifference subscales in community service and volunteering activities. When adolescents are exposed to diverse social issues by participating in service-learning programs, they can obtain hands-on experience, compare the compassion scores between pre- and posttests, and gradually be equipped with a spirit of caring. At the macro level, local policymakers can implement targeted strategies to foster compassion, social responsibility, and community engagement by identifying factors that facilitate or inhibit compassion with the CS validated in this study.

Our study posed some noteworthy limitations. First, our findings may only apply to Hong Kong adolescents due to the historical influence in the territory. More investigations should be conducted among different populations. Second, this study used cross-sectional data, but longitudinal research designs could be used in future studies to examine the temporal stability of this operationalization. In the future, the relationships with key indicators of psychological well-being and mental health can be included. For example, using cross-lagged structural equation modeling analysis, we can track how social connectedness and self-efficacy interact with compassion in the long run among adolescents. Moreover, we can explore the relationship between compassion and self-compassion among adolescents to design tailor-made intervention plans to promote their mental health.

## Conclusion

In conclusion, this study has demonstrated the construct validity and reliability of the 16-item CS among Hong Kong adolescents. It has examined its applicability in a cultural context deeply influenced by Confucianism, Western individualism, and neoliberal ideals, which is quite different from those contexts covered in previous studies. The findings provide robust support for the CS as a reliable and valid measure for cross-cultural research on compassion and yield evidence-based implications for compassion interventions.

## Data Availability

The datasets presented in this article are not readily available because the data are not publicly available due to privacy or ethical restrictions. The data that support the findings of this study are available on request from the corresponding author. Requests to access the datasets should be directed to syngai@cuhk.edu.hk.
