# Supplementary material for: Compassion Scale: factor structure and scale validation in Hong Kong adolescents
Source: Front Psychol. 2025 Feb 26;16:1508402. doi: 10.3389/fpsyg.2025.1508402 (PMC11897485; doi:10.3389/fpsyg.2025.1508402)
Supplement: Supplementary file 1 [file Supplementary_file_1.docx]

**Appendixes**

**Appendix I** Item and reliability test

|  | | Total Sample | | Subsample 1 | | Subsample 2 | |
| --- | --- | --- | --- | --- | --- | --- | --- |
|  |  | Corrected item-dimension correlation | Cronbach's alpha | Corrected item-dimension correlation | Cronbach's alpha | Corrected item-dimension correlation | Cronbach's alpha |
| Compassion | | / | 0.904 | / | 0.898 | / | 0.916 |
| Benevolence | M1 | 0.700 | 0.904 | 0.664 | 0.889 | 0.733 | 0.918 |
|  | M2 | 0.701 |  | 0.678 |  | 0.726 |  |
|  | M3 | 0.703 |  | 0.657 |  | 0.746 |  |
|  | M4 | 0.765 |  | 0.755 |  | 0.774 |  |
|  | K1 | 0.714 |  | 0.705 |  | 0.720 |  |
|  | K2 | 0.714 |  | 0.707 |  | 0.721 |  |
|  | K3 | 0.675 |  | 0.631 |  | 0.716 |  |
|  | K4 | 0.718 |  | 0.690 |  | 0.744 |  |
| Common Humanity | CH1 | 0.691 | 0.769 | 0.644 | 0.729 | 0.736 | 0.804 |
|  | CH2 | 0.670 |  | 0.633 |  | 0.703 |  |
|  | CH3 | 0.674 |  | 0.667 |  | 0.684 |  |
|  | CH4 | 0.660 |  | 0.617 |  | 0.699 |  |
| Indifference | I1 | -0.410 | 0.830 | -0.393 | 0.820 | -0.426 | 0.839 |
|  | I2 | -0.469 |  | -0.475 |  | -0.465 |  |
|  | I3 | -0.526 |  | -0.507 |  | -0.544 |  |
|  | I4 | -0.535 |  | -0.482 |  | -0.585 |  |

**Appendix II** Composite reliability and average variance extracted

|  | Composite Reliability | | Average Variance Extracted | |
| --- | --- | --- | --- | --- |
|  | Total Sample | Subsample 2 | Total Sample | Subsample 2 |
| Benevolence | 0.907 | 0.919 | 0.551 | 0.589 |
| Common Humanity | 0.775 | 0.808 | 0.494 | 0.514 |
| Indifference | 0.831 | 0.840 | 0.552 | 0.568 |

**Appendix III** The 95% confidence intervals of the correlations between the factors

|  | Sub-sample 2 | | Total Sample | |
| --- | --- | --- | --- | --- |
|  | Correlation | 95% CI | Correlation | 95% CI |
| Benevolence & Common Humanity | 0.696 | [0.643, 0.746] | 0.675 | [0.653, 0.698] |
| Benevolence & Indifference | -0.320 | [-0.418, -0.253] | -0.335 | [-0.418, -0.253] |
| Common Humanity & Indifference | -0.443 | [-0.564, -0.404] | -0.484 | [-0.564, -0.404] |

**Appendix IV** The likelihood ratio test results

| Sample | Model | Log likelihood | *df* | AIC | BIC | $\chi^{2}$ | *p* |
| --- | --- | --- | --- | --- | --- | --- | --- |
| Total Sample | Unconstrained | -24786.08 | 35 | 49491.26 | 49669.21 | 150.90 | <0.001 |
|  | Constrained | -24710.63 | 33 | 49638.16 | 49805.94 |  |  |
| Subsample 2 | Unconstrained | -12190.70 | 35 | 24350.05 | 24503.71 | 101.36 | <0.001 |
|  | Constrained | -12140.03 | 33 | 24447.41 | 24592.29 |  |  |
